# The Role of Subjective Wellbeing in Mediating Social Trust to the Mental Health of Health Workers

**DOI:** 10.3390/healthcare11091327

**Published:** 2023-05-05

**Authors:** Change Xiong, Yanqiu Yao, Tong Hu, Jing Cheng, Shandan Xu, Chaojie Liu

**Affiliations:** 1 Hubei Province Key Laboratory of Occupational Hazard Identification and Control, Institute of Social Development and Health Management, School of Public Health, Wuhan University of Science and Technology, Wuhan 430065, China; ht0601@wust.edu.cn (T.H.); xushandan@wust.edu.cn (S.X.); 2 Hubei Province Key Laboratory of Occupational Hazard Identification and Control, Institute of Nursing Research, School of Medicine, Wuhan University of Science and Technology, Wuhan 430065, China; yaoyanqiu@wust.edu.cn; 3 School of Psychology and Public Health, La Trobe University, Melbourne, VIC 3086, Australia; c.liu@latrobe.edu.au

**Keywords:** social trust, mental health, subjective wellbeing, health worker, mediating effect

## Abstract

Mental health problems of health workers are attracting increasing concerns in China and the world. A trustful relationship between health workers and patients is the foundation of quality patient care, which is currently under serious threat. This study aimed to determine the associations of social trust on subjective wellbeing and mental health of health workers. Using the survey data of 262 health workers extracted from the 2018 Chinese Family Panel Studies, a structural equation model with partial least square approach was established. The results showed that social trust was linked to both subjective wellbeing (β = 0.251, *p* < 0.01) and mental health (β = −0.210, *p* < 0.01). The effect of social trust on mental health was partially mediated by subjective wellbeing (51.87%). The association between social trust and subjective wellbeing was moderated by socioeconomic status: social trust has a stronger effect on subjective wellbeing in those with higher socioeconomic status. Erosion of social trust may present a serious risk to mental health and subjective wellbeing of health workers. High socioeconomic status can amplify the effect of social trust.

## 1. Introduction

Mental health problems of health workers are attracting increasing attention all over the world. High levels of work stress are common in health professionals [1]. Psychological disorders caused by work stress can affect cognitive functions such as concentration, understanding, and decision-making [2], leading to poor quality of patient care [3,4]. Maintaining mental health of health workers is also critical in response to the COVID-19 pandemic [5]. Subjective wellbeing is a crucial indicator impacting the mental health of health workers [6]. However, subjective wellbeing of Chinese health workers had a decreasing trend from 2004 to 2020 [7]. Previous research has established important positive relationships between social trust and subjective wellbeing [8]. Trustful relationships with family and relatives, friends, and neighbors are considerably related to subjective wellbeing [9]. Doctor–patient trust as a special kind of trust relationship has become discordant in recent years. Violent conflicts between doctors and patients have caused burnout syndrome and emotional exhaustion of health workers in China [10].

Health workers in China are facing unprecedented challenges in managing their relationships with patients, which has put a great deal of stress on them [3]. Since the introduction of the market mechanism in China [11], health workers have been charged with a responsibility to generate revenues through user charges for their institutions because of the declined share of governmental investment in the 1990s [12]. This has led to a public outcry on the affordability of medical care, causing a growing tension between health workers and consumers. Meanwhile, work pressure on health workers has been increasing, partly due to insufficient resources and partly due to increased consumer demands [13,14]. For example, China has 3.34 nurses per 1000 population, compared with 3.69 on average in the world, 8.34 in the US, and 7.93 in European countries [15]. There have been increasing concerns in recent years over the high prevalence of work stress and burnout in health workers in China [16]. Meanwhile, the mismatch between supply and demand has exacerbated the tension between health workers and consumers [17], eroding social trust from the public.

Social trust falls into the category of systemic trust, which refers to the expectation that individuals and groups can rely on others and the overall institutional system of the society for their interest [18]. According to Putnam [19], social trust is a manifestation of social capital. There is a paucity in the literature documenting social trust from health workers, despite speculation on its association with mental health [20]. Health workers are often expected to take social responsibilities such as fight against disease outbreaks in addition to their individual-based clinical works [21,22]. However, the deterioration of public trust in health workers is likely to jeopardize the trust of health workers in others [23], which can trigger a sense of doubt about the public appreciation for their contributions [24]. Previous studies in community settings have shown that low levels of social trust are associated with poor self-rated health [25,26].

**Figure 1:**
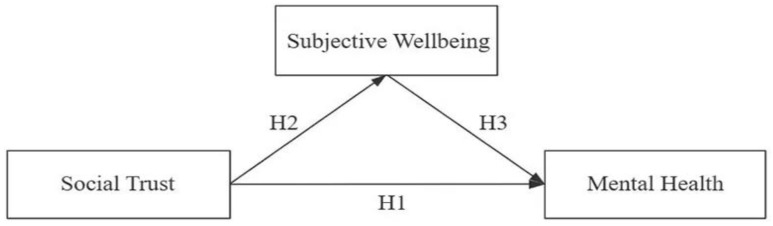
Hypotheses tested in the study.

This study aimed to address the gap in the literature by assessing social trust held by health workers and testing its associations with subjective wellbeing and mental health. Four hypotheses were proposed (Figure 1):

**Hypothesis 1 (H1).**  *Higher social trust is associated with better mental health.*

**Hypothesis 2 (H2).**  *Higher social trust is associated with higher subjective wellbeing.*

**Hypothesis 3 (H3).**  *Higher subjective wellbeing is associated with better mental health.*

**Hypothesis 4 (H4).**  *Subjective wellbeing mediates the effect of social trust on mental health.*

### 1.1. Review of the Literature and Hypothesis Development

#### 1.1.1. Relationship between Social Trust and Mental Health

Previous studies have demonstrated a positive correlation between social trust and general health [27]. This aligns well with the theory of social determinants of health [28]. It is worth noting that social trust is not only important for individual health and wellbeing but also has an impact on various aspects of the healthcare system. Social trust encourages cooperative behaviors, social cohesion, social solidarity, and collective actions [29]. Empirical evidence shows that community residents who trust and help each other have better mental health than those who do not [30,31,32]. Social trust can help with the healing process in those who experience traumatic events [33], mitigate the negative mental health consequences of socioeconomic deprivations on children [34], and ease psychological distress of older people [35]. Low levels of trust in unknown people are associated with high levels of mental stress [36]. Research also suggests that poor mental health has a significant negative impact on social trust [37].

#### 1.1.2. Relationship between Social Trust and Subjective Wellbeing

Subjective wellbeing reflects an individual’s affirmative attitudes and positive feelings arising from a comparison between the actual state of life and the ideal state of life [38]. Life satisfaction is one of the key elements of subjective wellbeing [39]. Many external factors such as event context and demographics can affect subjective wellbeing [40].

A growing body of the literature points to a positive connection between social trust and subjective wellbeing. Trustful relationships with family members, relatives, friends, and neighbors are more closely linked to subjective wellbeing than structural social capital [9,19]. Social trust has been found to be beneficial to the emotional wellbeing of rural people in China [41], possibly through the pathway of social networking and social support [42]. A survey of 1449 left-behind children (who did not live with their emigrated parents) in China found that social cohesion and trusting relationships with caregivers positively predict the subjective wellbeing of the left-behind children [43]. To the best of our knowledge, there has been no research into the association between social trust and subjective wellbeing of health workers.

#### 1.1.3. Relationship between Subjective Wellbeing and Mental Health

The link between subjective wellbeing and mental health has been well documented. High levels of subjective wellbeing are associated with good health, longevity, good social relationships, high job performance, and creativity [44]. Previous studies have confirmed that the association between subjective wellbeing and health varies by age [45], but overall, high levels of subjective wellbeing are associated with lower levels of mental health problems, such as stress, depression, anxiety, and loneliness [46]. Subjective wellbeing is also closely related to long-term health behaviors [47].

#### 1.1.4. Relationships between Social Trust, Subjective Wellbeing, and Mental Health

Given that social trust is linked to both subjective wellbeing and mental health, a mediation analysis is warranted to examine the role of subjective wellbeing in the association between social trust and mental health. A previous study showed that high subjective wellbeing can effectively reduce the impact of social trust (or a lack of) on the emotional health of older people [41]. Institutional trust has been proved to partially mediate the relationship between subjective wellbeing and mental health [48]. Social trust also partially mediates the effect of satisfaction with social security on subjective wellbeing [49].

## 2. Materials and Methods

### 2.1. Data Source

Data were extracted from the 2018 China Family Panel Studies (CFPS), which are publicly available. No ethics approval was required.

The CFPS was started in 2010 by the Institute of Social Science Survey (ISSS) at Peking University. It aims to reflect the changes of China’s society, economy, population, education, and health by tracking and collecting data at the individual, family, and community levels and focuses on the economic and non-economic wellbeing of Chinese residents, including economic activities, educational outcomes, family relations and dynamics, population migration, and health. There are four main types of CFPS questionnaires: community questionnaire, family questionnaire, adult questionnaire, and children questionnaire. Adult questionnaires were used in this study. Five waves of the survey have been completed since then, with samples drawing from 25 provinces/regions in mainland China. A probability proportional to size (PPS) sampling strategy was employed to recruit study participants. Data were collected through computer-assisted personal interviews, followed by quality audit measures such as random telephone and field checks, audio recording, interview reviews, and logical analyses [50].

We used the 2018 CFPS dataset for this study because it contains the largest sample of our target participants (health workers). The 2018 CFPS collected 37,354 records, in which 281 were completed by respondents flagged as a physician, a nurse, or an allied health professional. We adopted imputation method strategy to manage missing values. This resulted in a final sample of 262 participants for data analyses.

### 2.2. Measurements

#### 2.2.1. Mental Health

Mental health was the outcome indicator in this study. It was measured using the simplified Epidemiological Studies-Depression Scale (CES-D8), which has demonstrated good reliability and validity in China [51]. In this study, 5 items were selected as outer loadings that are more than 0.65 (Appendix A Table A1). Respondents were asked to report how often in the past week they felt depressed, struggled, felt happy (score reversed), felt joyful (score reversed), and felt sad, along a scale ranging from 0 ‘none of the time’ to 3 ‘almost or all of the time’. A summed score (0–15) was calculated, with a higher score indicating worse mental health.

#### 2.2.2. Social Trust

The effect of social trust on mental health was the major interest of this study. In the CFPS, social trust was defined as a belief in the honesty, integrity, and reliability of other people with whom one usually interacts in daily life [52]. Respondents were asked to rate their trust in parents, Americans, strangers, neighbors, physicians, and local governmental officials, respectively, along a ten-point scale ranging from 0 (strongly distrust) to 10 (strongly trust) [53]. In this study, three items (neighbors, physicians, and local governmental officials) were selected to measure social trust as outer loadings are more than 0.65 (Appendix A Table A1). A summed score (0–30) was calculated, with a higher score indicating a higher level of social trust.

#### 2.2.3. Subjective Wellbeing

This study tested the mediating effect of subjective wellbeing on the association between social trust and mental health. Subjective wellbeing was measured based on the concept proposed by Diener [54], encompassing life satisfaction, life happiness, and future self-confidence. Diener [54] identified three features of subjective wellbeing: subjectivity, relative stability, and integrity. Respondents were asked to rate their life satisfaction and future self-confidence on a five-point Likert scale, while life happiness was rated on a ten-point scale ranging from 1 (very unhappy) to 10 (very happy). A summed score (0–20) was calculated, with a higher score indicating a higher level of subjective wellbeing.

#### 2.2.4. Living and Working Conditions

In this study, we also tested the association between sleep time and subjective wellbeing and the moderation effect of socioeconomic status (SES) on the association between social trust and subjective wellbeing. Healthcare services often require night shifts, which can disrupt daily routines, leading to low levels of subjective wellbeing [55]. In this study, sleep time was measured by a latent variable containing two items: average daily sleep hours over workdays and average daily sleep hours over non-workdays.

Empirical evidence shows that people with low SES tend to have low social trust and poor health outcomes [56,57]. A previous study found that higher levels of public health investment appear to be associated with both higher levels of subjective wellbeing and higher levels of health outcomes [58]. In this study, SES was measured by a latent variable containing three items: average annual household income (<50,000, 50,000–100,000, >1,000,000 Yuan), individual educational attainment (with or without a university degree), and self-rated social status. Traditional SES measures usually cover income, education, and occupation [59,60]. However, self-perceived social status is a much stronger predictor of health outcomes than occupation [57]. In the CFPS, respondents were asked to rate their social status in comparison with others in the local community on a five-point scale ranging from 1 (very low) to 5 (very high), which was collapsed into three categories in data analyses: low (1–2), average (3), and high (4–5).

#### 2.2.5. Sociodemographic Characteristics

Data in regard to age (≤35, 36–64, ≥65 years), sex (male or female), household registration (urban or rural), marital status (never married, married/cohabiting, or widowed/divorced), and profession (physician, nurse, or allied health profession) were collected in the 2018 CFPS.

### 2.3. Statistical Analysis

Data were analyzed using SPSS 26.0 and Smart PLS 4.0.

The sociodemographic characteristics of study participants were described using frequency distributions. The mean values and standard deviations (SDs) of social trust, mental health, and subjective wellbeing of the study participants with different sociodemographic characteristics were calculated and compared through student *t* tests or ANOVA.

Structural equation modeling with partial least square (PLS-SEM) was established to test the study hypotheses. We chose PLS-SEM because it does not require a large sample size nor a normal distribution of data [61,62]. The modeling started with a testing of reliability and validity of the key constructs measured. Reliability was assessed using Cronbach’s α, rho-A, and composite reliability, with a higher than 0.6 coefficient deemed acceptable [63]. Validity was assessed through convergent validity and discriminant validity. The former was reflected by the average variance extracted (AVE), with a minimum threshold value of 0.5 [64]. The latter was assessed through the Fornell–Larker criterion and heterotrait–monotrait ratio of correlations (HTMT) [65,66]. The Fornell–Larcker criterion requires that the square root of each AVE is higher than the correlation coefficients between the tested construct and other constructs [67]. The HTMT ratio measures similarity between tested constructs, which must be lower than 0.90 [65,68,69].

Once the reliability and validity of the tested constructs were confirmed, PLS-SEM was established using a 5000 bootstrapped procedure [68]. Fitness of data into the model was assessed through standardized root mean square (SRMR between 0 and 1) [67], R^2^ (between 0 and 1), Q^2^ (>0) [65], and root mean squared error (RMSE) and mean absolute error (MAE) with a naïve benchmark [69]. While R^2^ and Q^2^ reflect in-sample explanatory power of the model, RMSE and MAE indicate out-of-sample predictive power of the PLS path model estimations [69]. The naïve benchmark for RMSE and MAE was generated through linear regression modelling (LM). A high predictive power is assumed if none of the constructs in the PLS-SEM have a higher RMSE or MAE value compared to the naïve LM benchmark [69,70].

A two-side *p* value less than 0.05 was considered statistically significant for the path coefficients. We calculated the VAF (variance accounted for) value to determine the mediation effect: less than 20% indicates no mediation, 20–80% indicates partial mediation, and above 80% indicates full mediation [64].

## 3. Results

### 3.1. Characteristics of Respondents

The majority of study participants were female (67%), registered as an urban resident (52%), and married or cohabited with others (78%) at the time of the survey. About half were younger than 35 years. Most participants were physicians (40%) or nurses (40%) and have a university degree (70%). The average sleep time was 7.28 (SD = 1.36) hours on workdays and 7.94 (SD = 1.55) hours on non-workdays. Over 43% of participants reported an annual household income between 50,000 and 100,000 Yuan. About 58% considered their local social status as average (Table 1).

Marriage was associated with higher levels of social trust (*p* = 0.003), mental health (*p* = 0.001), and subjective wellbeing (*p* = 0.025). Higher self-rated social status was associated with higher levels of social trust (*p* = 0.023) and subjective wellbeing (*p* = 0.006). Nurses had the lowest level of social trust (*p* = 0.029) (Table 1).

The study participants reported a mean score of 18.65 (SD = 4.45), 8.95 (SD = 2.68), and 15.49 (SD = 3.02) for social trust, mental health, and subjective wellbeing, respectively, similar to those of non-health workers (Appendix A Table A2).

### 3.2. PLS-SEM Results

The PLS-SEM demonstrated acceptable reliability and validity of key constructs as indicated in Table 2, Table 3 and Table 4. The reliability coefficients of mental health and subjective wellbeing were all above 0.7, despite a slightly lower but still acceptable Cronbach’s alpha and Rho-A for social trust (>0.6 in Table 2) possibly due to its low number of measurement items [64]. The AVE values for the three constructs were all above 0.5 (Table 2), demonstrating acceptable convergent validity. The discriminant validity of the three constructs was supported by the Fornell–Larcker criterion (Table 3) and the HTMT ratio (Table 4).

Overall, the PLS-SEM showed good fitness of data: SRMR = 0.085; R^2^ = 0.286; Q^2^ = 0.138. The path estimations showed medium predictive power, with most RMSE and MAE values being higher than their naïve LM benchmarks (Table 5).

**Figure 2:**
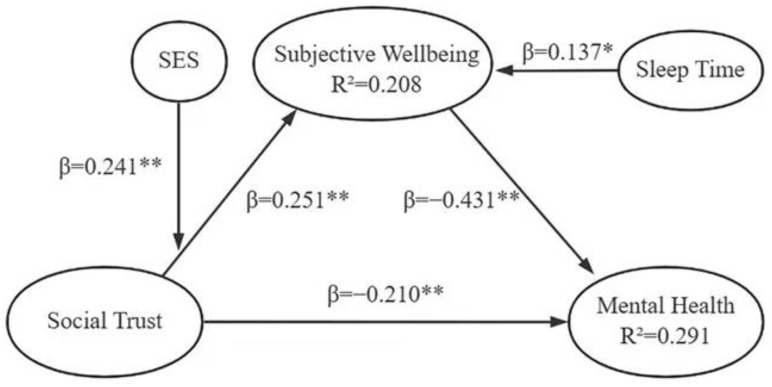
Social trust, subjective wellbeing, and mental health: PLS-SEM. Note: * *p* < 0.05, ** *p* < 0.01.

The PLS-SEM indicated a direct link between social trust and mental health: one unit increase in social trust was associated with 0.210 units of improvement in mental health (a reduction of mental health problems) (Figure 2): hypothesis one (H1) was accepted. One unit increase in social trust was also associated with a 0.251 unit increase in subjective wellbeing (Figure 2): hypothesis two (H2) was accepted. One unit increase in subjective wellbeing was associated with 0.431 units of improvement in mental health (a reduction in mental health problems) (Figure 2): hypothesis three (H3) was accepted.

Subjective wellbeing partially mediated the effect of social trust on mental health: the indirect effect accounted for 51.87% of the variance (Table 6). Hence, hypothesis four (H4) was accepted.

Further analyses showed that longer sleep duration was associated with higher subjective wellbeing (β = 0.173, *p* = 0.026). SES moderated the effect of social trust on subjective wellbeing: higher effect of social trust on subjective wellbeing was found in those with higher SES (β = 0.241, *p* = 0.048).

## 4. Discussion

Health workers have similar levels of social trust, subjective wellbeing, and mental health in comparison with their non-health counterparts in China, according to the findings of this study. There exist significant links among these three constructs in health workers. Firstly, higher social trust is associated with better mental health. This result is consistent with the findings of previous studies [31,64,71,72]. Social trust may reduce work stress of health workers through a perception of supportive work and social environments. Public and consumer engagement has been considered as a critical determinant of patient care outcomes. Social trust provides a fundamental condition for effective consumer engagement. Higher levels of social trust can make health workers feel psychologically safe to work in partnerships with their patients to achieve clinical excellence [23,73], reducing psychological distress [74].

Secondly, higher social trust is associated with higher subjective wellbeing. This finding is supported by several other studies [19,43]. The Chinese culture emphasizes kinship and “Guanxi” [75]. Those who have a large network of social connections are highly regarded and respected, and they tend to have higher levels of life satisfaction. Social connection from family members, friends, coworkers, and other adults is a known protective factor for mental health. Family and friend relationships make unique contributions to wellbeing [76,77]. High levels of social trust enable people to enjoy their daily life, reducing negative emotions such as a hostile mentality, anger, and anxiety. The association between social trust and subjective wellbeing may be bidirectional [78]. Social trust encourages people to develop and expand ties with others, increasing subjective wellbeing. Meanwhile, subjective wellbeing helps foster more trustful relationships with others. Trust is essential for health workers to maintain good relationships with patients. A study of physicians in China showed a clear connection between public trust and self-rated happiness [79]. However, public trust in health workers has been declining in China in recent years. Meanwhile, health workers have reported lower levels of social trust in comparison with their patients [80,81,82].

Thirdly, higher subjective wellbeing is associated with better mental health. This is not surprising as happiness, one of the elements of subjective wellbeing, is an indication of healthy emotion. A study of 3989 adults in Australia reported that subjective wellbeing reduces vulnerability to depression and anxiety [83]. Another study during the outbreak of COVID-19 found that lower subjective wellbeing of frontline health workers is associated with moral injury, burnout, and psychological distress [81]. In China, physicians perceived high levels of stress, burnout, and a declined sense of wellbeing as indications of poor workplace wellbeing [82]. Health workers maybe have a low level of subjective wellbeing in high stress scenarios. However, our study showed there was not significant difference between subjective wellbeing of physicians under normal working conditions and the general public. A more complex mechanism needs to be further explored.

Fourthly, subjective wellbeing partially mediates the effect of social trust on mental health. The result is consistent with the findings of previous studies of young and middle-aged adults [84,85]. The psychological burden of health workers can be exacerbated when subjective wellbeing is at a low level. Health workers are constantly exposed to stressful events such as the outbreak of COVID-19, which would undoubtedly affect their subjective wellbeing. Working under a stressful environment influences the everyday life of health workers [86]. It is important to pay increasing attention to the subjective wellbeing of health workers.

The moderation role of SES in the link between social trust and subjective wellbeing revealed in this study warrants further studies. We found a stronger effect of social trust on subjective wellbeing in those with higher SES. The underlying mechanism of such a moderation effect is unclear, but it highlights the challenges for improving the mental health of people who have a lower perceived SES. SES has been found to be a strong predictor of health outcomes [87]. Some researchers argue that poverty alleviation and wealth increase should be taken as a top priority for improving the wellbeing of people [88,89]. However, this is not enough. Individual perception of SES often involves a comparison with others. Internationally, there exists a hierarchical structure in health professionals, with medical doctors usually enjoying the top status. The vast majority of health workers such as nurses may rate their SES as lower in comparison with medical doctors. In China, health workers of tertiary hospitals often have higher qualifications and higher income and attract higher social trust than their counterparts in the primary care sector [90]. This can lead to a vicious cycle, making mental health promotion for nurses and primary care workers extremely challenging.

Sleep deprivation has been an occupational health concern for health workers [91]. We found a direct link between sleep time and subjective wellbeing. Previous studies show that health workers working long night shifts suffer higher incidence of depression, stress, and burnout [92]. Sleep latency mediates the effect of night-shift work on mental wellbeing [93]. Sleep disturbance is often associated with poor job performance, poor doctor-patient relationship, and psychosomatic symptoms [94]. Poor quality of sleep can mediate the effect of anxiety on subjective wellbeing [95].

## 5. Conclusions

This research revealed that the social trust and subjective wellbeing play a substantial role in promoting the mental health of health workers. Thus, it can be recommended that improving the level of social trust especially interpersonal trust is an important strategy to promote the mental health of health workers. On the contrary, erosion of social trust may present a serious risk to mental health and subjective wellbeing of health workers. Health service managers can foster a culture and service environment, in which health workers feel safe, supported, and protected. They can also promote a patient safety culture that discourages status distinction across health professions. Increasing policy attention should be paid to pay equality and work–life balance for health workers. These measures are essential for promoting the mental health and subjective wellbeing of health workers and for ensuring the safety and quality of patient care.

## 6. Limitations and Future Research

The present study has several limitations. The data used in this study were drawn from a Chinese context. The effect of social trust on mental health workers is likely to vary under different system contexts. We were not able to explore the occupational differences of the effect of social trust on mental health either due to limited sample size. Our study adopted a cross-sectional design, and causal conclusions should not be assumed.

## Figures and Tables

**Table 1:** Sociodemographic characteristics, social trust, mental health, and subjective wellbeing of study participants.

Characteristics		*n*	%	Mean ± Standard Deviation (SD)		
				Social Trust	Mental Health	Subjective Wellbeing
Sex	Male	87	33.20	18.25 ± 4.692	9.09 ± 2.783	15.49 ± 3.005
	Female	175	66.80	19.48 ± 4.459	8.68 ± 2.451	15.49 ± 3.162
				*p* = 0.424	*p* = 0.926	*p* = 0.786
Age (Years)	≤35	130	49.62	18.33 ± 4.836	8.86 ± 2.563	15.85 ± 2.741
	36–65	61	23.28	18.92 ± 4.591	9.34 ± 2.750	15.03 ± 3.077
	≥65	71	27.10	19.03 ± 4.342	8.77 ± 2.829	15.23 ± 3.506
				*p* = 0.796	*p* = 0.191	*p* = 0.026
Profession	Physician	106	40.46	19.32 ± 4.174	8.73 ± 2.572	15.34 ± 2.995
	Nurse	106	40.46	17.62 ± 4.994	9.36 ± 2.792	15.54 ± 3.246
	Allied health	50	19.08	19.44 ± 4.496	8.52 ± 2.589	15.70 ± 2.779
				*p* = 0.029	*p* = 0.702	*p* = 0.309
Household registration	Urban	124	47.33	18.62 ± 4.383	9.04 ± 2.712	15.48 ± 2.804
	Rural	138	52.67	18.69 ± 4.882	8.86 ± 2.657	15.50 ± 3.270
				*p* = 0.266	*p* = 0.719	*p* = 0.589
Marital status	Never married	48	18.32	17.23 ± 5.203	8.68 ± 2.784	15.06 ± 0.392
	Married/cohabiting	205	78.24	19.16 ± 4.370	8.90 ± 2.581	15.62 ± 2.856
	Widowed/divorced	9	3.44	14.78 ± 4.684	11.33 ± 3.464	14.69 ± 5.099
				*p* = 0.003	*p* = 0.001	*p* = 0.025
Annual household income (Yuan)	<50,000	60	22.90	17.65 ± 4.410	8.95 ± 2.936	15.62 ± 3.552
	50,000–100,000	115	43.89	19.42 ± 4.680	8.83 ± 2.588	15.43 ± 3.115
	>100,000	87	33.21	15.48 ± 2.696	9.10 ± 2.637	15.48 ± 2.596
				*p* = 0.702	*p* = 0.201	*p* = 0.159
Qualification	university degree	182	69.50	18.41 ± 4.700	9.08 ± 2.758	15.24 ± 3.245
	Under University degree	80	30.50	19.23 ± 4.492	8.65 ± 2.480	16.05 ± 2.490
				*p* = 0.817	*p* = 0.197	*p* = 0.156
Self-rated social status	Low	55	20.30	16.24 ± 4.554	9.67 ± 2.956	13.85 ± 3.778
	Average	153	58.39	18.55 ± 3.834	8.97 ± 2.714	15.55 ± 2.688
	High	54	20.61	21.43 ± 4.087	8.14 ± 2.031	16.98 ± 2.359
				*p* = 0.023	*p* = 0.314	*p* = 0.006

**Table 2:** Reliability of measurement constructs.

Construct	Outer Loading	Cronbach’s α	Rho-A	Composite Reliability	AVE
Social trust (ST)		0.600	0.620	0.781	0.545
ST-1	0.794				
ST-2	0.713				
ST-3	0.703				
Subjective wellbeing (SWB)		0.725	0.749	0.842	0.640
SWB-1	0.830				
SWB-2	0.796				
SWB-3	0.774				
Mental health (MH)		0.761	0.764	0.839	0.511
MH-1	0.710				
MH-2	0.715				
MH-3	0.720				
MH-4	0.737				
MH-5	0.689				

**Table 3:** Discriminant validity—Fornell–Larcker criterion.

Constructs	ST	SWB	MH
Social trust (ST)	0.738		
Subjective wellbeing (SWB)	0.342	0.800	
Mental health (MH)	−0.357	−0.502	0.714

Note: Diagonal values are the square root of the AVE values of each respective construct.

**Table 4:** Discriminant validity—Heterotait–Monotrait (HTMT) ratio.

Constructs	ST	SWB	MH
Social trust (ST)	-		
Subjective wellbeing (SWB)	0.488	-	
Mental health (MH)	0.507	0.650	-

**Table 5:** Predictive results of partial least square (PLS) and linear regression modeling (LM).

	Root Mean Squared Error (RMSE)		Mean Absolute Error (MAE)		Q^2^	
	LM	PLS	LM	PLS	LM	PLS
MH-1	0.672	0.678	0.559	0.559	0.079	0.067
MH-2	0.614	0.666	0.530	0.562	0.014	0.095
MH-3	0.682	0.856	0.561	0.707	0.054	0.051
MH-4	0.833	0.823	0.691	0.689	0.025	0.050
MH-5	0.854	0.597	0.716	0.519	0.055	0.068
SWB-1	0.819	1.856	0.622	1.409	0.070	0.171
SWB-2	0.828	0.823	0.633	0.630	0.039	0.060
SWB-3	1.833	0.815	1.414	0.628	0.191	0.070

**Table 6:** Results of hypothesis testing.

Hypothesis	Path	Path Coefficient	95% CI		f^2^	T	*p*	Decision
H1	SC→MH	−0.210	−0.323	−0.096	0.055	3.599	<0.001	Accepted
H2	SC→SWB	0.251	0.145	0.361	0.070	4.488	<0.001	Accepted
H3	SWB→MH	−0.434	−0.547	−0.316	0.231	7.331	<0.001	Accepted
Mediation analysis	Path		Indirect effect			Total effect	Variation accounted for (VAF)	Mediation effect
H4	SC→SWB→MH		−0.109			−0.210	0.5187	Partial

## Data Availability

The datasets generated during the current study are available in the Peking University Open Research Data Platform repository, https://opendata.pku.edu.cn/dataset.xhtml?persistentId=doi:10.18170/DVN/45LCSO (accessed on 15 June 2020).

## Appendix A

**Table A1:** Outer loadings of measurement constructs.

Construct	Outer Loadings
Social trust (ST)	
ST-1: Trust in neighbors.	0.747
ST-2: Trust in local government officials.	0.658
ST-3: Trust in physicians.	0.628
ST-4: Trust in parents.	0.443
ST-5: Trust in Americans.	0.497
ST-6: Trust in strangers.	0.527
Subjective wellbeing (SWB)	
SWB-1: What is your happiness level?	0.843
SWB-2: How satisfied are you with your life?	0.788
SWB-3: How confident do you feel about the future?	0.762
Mental health (MH)	
MH-1: I felt depressed.	0.713
MH-2: I struggled.	0.740
MH-3: I felt delighted.	0.664
MH-4: I felt joyful in life.	0.658
MH-5: I felt sad.	0.715
MH-6: I did not sleep well.	0.583
MH-7: I felt lonely.	0.648
MH-8: I felt life could not go on.	0.460

**Table A2:** Social trust, mental health, and subjective wellbeing of health workers and non-health workers.

Participants	Sample Size (*n*)	Social Trust (Mean ± SD)	Mental Health (Mean ± SD)	Subjective Wellbeing (Mean ± SD)
Health workers	262	18.65 ± 4.64	8.95 ± 2.68	15.49 ± 3.05
Non-health workers	18199	18.53 ± 5.59	9.06 ± 2.86	15.63 ± 3.33
*p*		<0.001	0.395	0.676
